# 

**Published:** 1886-10-30

**Authors:** 


### THE HOSPITAL

An Institution, Family, and Parochial Journal of Hospitals, Asylums, & all Agencies for the Care of the Sick, Criticism & News.

### CONTENTS.

Good Work Done, and to be Done - - - - - 69
The Hospitals of our Grandfathers - - - - 70
Discharged. Not where to Lay his Head. By George Fleming. Chapter VI - - - - - - 71
The Registration Fee System. By the Right Hon. Lord Leigh - - - - - - - - - - 73
Words of Consolation.—The Discipline of Suffering - 74
Hospital Administration.—Alcohol at certain General and Special Metropolitan Hospitals, 1875-6 to 1885 - - 75
Notes and News.—The Registration Fee System.—Cottage Hospitals.—New Children's Hospital.—Statue of Sister Dora.—Out-patients' Subscription Boxes.—Miller Hospital at Greenwich.—Vacancies.—Sheffield School of Medicine.—Cremation Society of England.—Church Parades.—New Chapel for Dublin.—The Bishop of Durham and the Newcastle Infirmary.—The Highgate Infirmary.—Professor Dyce-Davidson.—Mr. Emil Behnke.—Doctor Collie on Enteric Fever.—Temporary Hospitals in Liverpool, etc., etc. - - - - 76
Annotations.—The Out-Patient Question.—The Exhibition of Competition Plans.—Incurable and Chronic *v.* Acute Diseases - - - - - - - - 79
Infectious Fevers and Diseases in London - - 80
From a Hospital for the Insane: The Few November Flowers - - - - - - - - 81
Reviews and Notices.—Convalescent Homes for Asylum Patients.—A Handbook on Nursing.—A Manual of Urine Testing - - - - - - - 81
Editor's Letter Box.—Invalid Passengers on Board Ship.—Nurses' and Probationers' Food.—Fruit for Hospitals - 82
Difficult Cases - - - - - - - 83
The Hospital Charity Box. Prizes and Results - - 83
The Children's Corner. Puzzles, Answers, etc. - - 84
The In-Patients' Hospital Diary: Special Hospitals - 85

